# Evaluating the Integration of One Health in Surveillance Systems for Antimicrobial Use and Resistance: A Conceptual Framework

**DOI:** 10.3389/fvets.2021.611931

**Published:** 2021-03-24

**Authors:** Cécile Aenishaenslin, Barbara Häsler, André Ravel, E. Jane Parmley, Sarah Mediouni, Houda Bennani, Katharina D. C. Stärk, David L. Buckeridge

**Affiliations:** ^1^Centre de recherche en santé publique de l'Université de Montréal et du CIUSSS du Centre-Sud-de-l'Île-de-Montréal, Montreal, QC, Canada; ^2^Research Group on Epidemiology of Zoonoses and Public Health, Faculty of Veterinary Medicine, Université de Montréal, Saint-Hyacinthe, QC, Canada; ^3^Veterinary Epidemiology Economics and Public Health Group, Department of Pathobiology and Population Sciences, Royal Veterinary College, Hatfield, United Kingdom; ^4^Centre for Foodborne, Environmental and Zoonotic Infectious Diseases, Public Health Agency of Canada, Guelph, ON, Canada; ^5^Department of Population Medicine, University of Guelph, Guelph, ON, Canada; ^6^Department of Animal Health, Federal Office for Food Safety and Veterinary Affairs, Bern, Switzerland; ^7^Department of Epidemiology, Biostatistics and Occupational Health, Faculty of Medicine, McGill University, Montreal, QC, Canada

**Keywords:** One Health, surveillance, evaluation, framework, methodology, antimicrobial resistance, integrated surveillance

## Abstract

It is now widely acknowledged that surveillance of antimicrobial resistance (AMR) must adopt a “One Health” (OH) approach to successfully address the significant threats this global public health issue poses to humans, animals, and the environment. While many protocols exist for the evaluation of surveillance, the specific aspect of the integration of a OH approach into surveillance systems for AMR and antimicrobial Use (AMU), suffers from a lack of common and accepted guidelines and metrics for its monitoring and evaluation functions. This article presents a conceptual framework to evaluate the integration of OH in surveillance systems for AMR and AMU, named the Integrated Surveillance System Evaluation framework (ISSE framework). The ISSE framework aims to assist stakeholders and researchers who design an overall evaluation plan to select the relevant evaluation questions and tools. The framework was developed in partnership with the Canadian Integrated Program for Antimicrobial Resistance Surveillance (CIPARS). It consists of five evaluation components, which consider the capacity of the system to: [1] integrate a OH approach, [2] produce OH information and expertise, [3] generate actionable knowledge, [4] influence decision-making, and [5] positively impact outcomes. For each component, a set of evaluation questions is defined, and links to other available evaluation tools are shown. The ISSE framework helps evaluators to systematically assess the different OH aspects of a surveillance system, to gain comprehensive information on the performance and value of these integrated efforts, and to use the evaluation results to refine and improve the surveillance of AMR and AMU globally.

## Introduction

Globally, antimicrobial resistance (AMR) is a major threat to public health (1). Although the development of resistant human pathogens is primarily driven by antimicrobial (AM) consumption in human populations, the use of AMs in animals also selects for resistant microorganisms, which can be transmitted to human through direct contact or through the food chain (2, 3). Moreover, residues of AM and/or resistant microorganisms are released into the ecosystem through waste from hospitals, municipalities, livestock and aquaculture farms, and manufacturing units (4–6). The spread of the existing resistant microorganisms and resistance genes, and the emergence of the new ones are major global concerns in the human and animal health sectors (7). Consequently, surveillance systems for AMR should integrate the surveillance of AMU and resistance in microorganisms circulating in humans, in animals, and in the environment (8, 9). This approach, in line with the concept of “One Health” (OH), is central to the global action plan of the WHO on AMR (10) and to the Food and Agriculture Organization (FAO)/Organization for Animal Health (OIE)/WHO tripartite collaboration on AMR (11). On a global scale, efforts have been made recently to develop guidance for the integrated surveillance of AMR, and several systems have been developed worldwide, with this goal, such as in Canada, Denmark, the Netherlands, Norway, South Korea, Sweden, and the United States (12). But despite years of experience with integrated surveillance in different countries, evidence of the added value of applying a OH approach is still lacking (13, 14).

The need for new knowledge about the effectiveness and economic efficiency of the integrated AMR surveillance systems has been underlined in Canada, where the Canadian Integrated Program for Antimicrobial Resistance Surveillance (CIPARS) has been in operation since 2002. CIPARS is a national program coordinated by the Public Health Agency of Canada (PHAC) which is dedicated to the collection, integration, analysis, and communication of trends in AMU and AMR in selected bacteria from humans, animals, and animal-derived food sources in Canada (15). The objectives of the CIPARS are to provide an integrated approach to monitor trends of AMU and AMR in humans and animals, to facilitate the assessment of the public health impact of AM used in humans and in the agricultural sectors, and to allow accurate comparisons with data from other countries that use similar surveillance systems. Although CIPARS is recognized as a leader in the development and implementation of highly integrated surveillance approaches for AMR at the human-animal-environment interface, the integrated approach used by the CIPARS has never been evaluated. Consequently, this study was undertaken in partnership with CIPARS and grew from their need to better evaluate the added value of an integrated OH approach for the surveillance of AMR and AMU in Canada.

An important challenge is that surveillance systems for AMU and AMR suffer from a lack of agreed-upon guidelines and metrics for monitoring and evaluating their integrated OH approach. This lack of standardization makes it challenging to compare and synthesize results across studies, contributing to the current lack of knowledge on the best OH surveillance strategies that should be promoted globally (16). Generic frameworks for the evaluation of surveillance systems that are commonly used, such as the CDC framework (17), do not include any guidance for the specific evaluation of OH integration into surveillance systems (18).

This article presents the Integrated Surveillance System Evaluation (ISSE) framework, a conceptual framework for evaluating the performance and value of OH integration in surveillance systems for AMU and AMR based on the Canadian experience. The main purpose of the ISSE framework is to assist the stakeholders and researchers in designing an overall evaluation plan for their integrated OH surveillance systems for AMU and AMR and in selecting the relevant evaluation questions. Specific methods and tools to address the evaluation questions are proposed and discussed. Finally, the complementarity of this approach and the relevance of other surveillance system evaluation tools are presented.

## Materials and Methods

**Figure 1:**
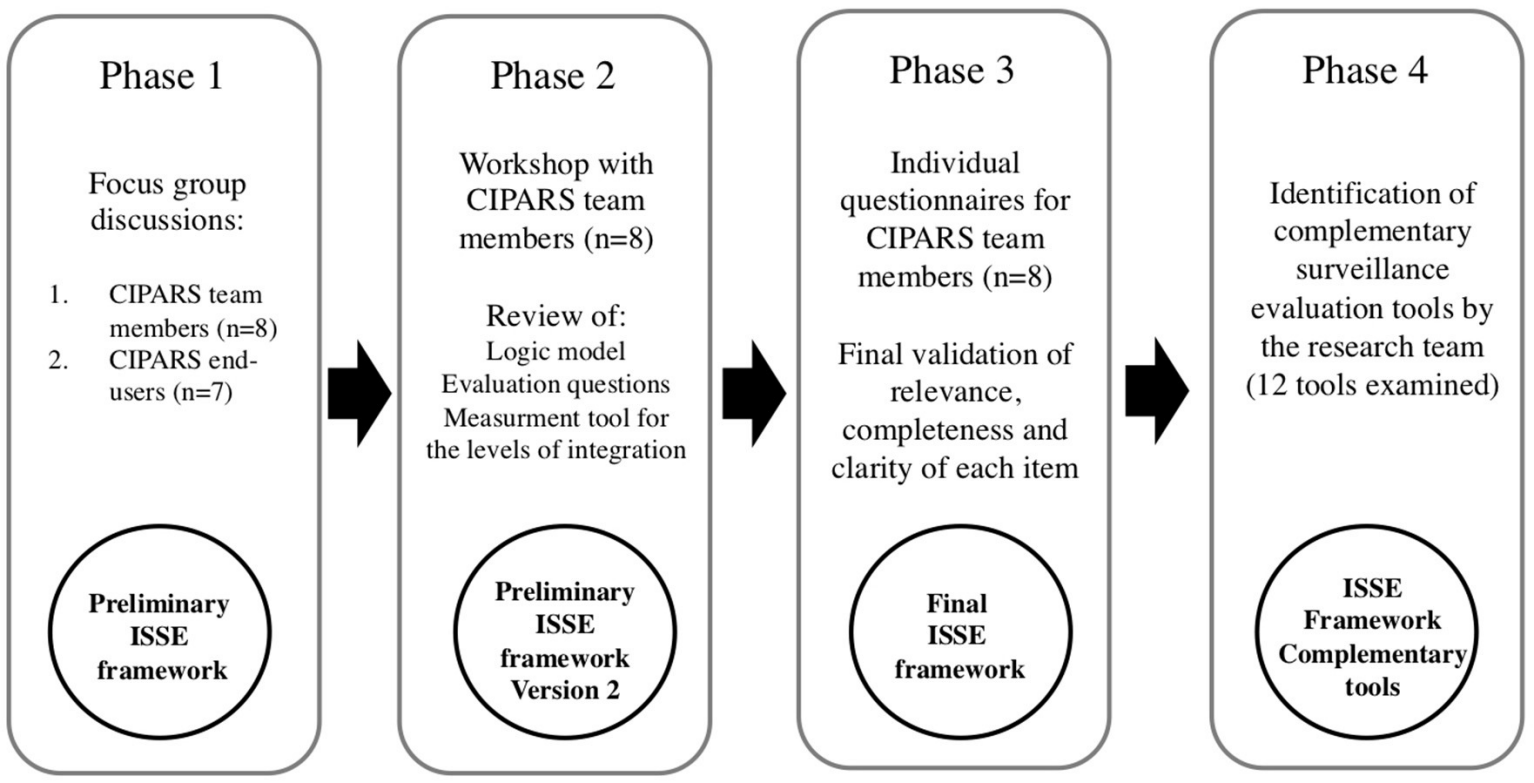
Methodological approach for the development of the ISSE framework.

The ISSE framework was developed using a participatory qualitative research design structured as a four-phase process (Figure 1). The study was conducted according to the ethical principles stated in the Declaration of Helsinki (2013). The ethical approval was obtained by the McGill Institutional Review Board (IRB Study Number A09-E61-16B). Written informed consent was obtained for all study participants before data collection.

### Phase 1: Focus Group Discussions

The first phase of the study collected information and perspectives prior to the development of the framework. Two focus group discussions (FGDs) were conducted with two categories of participants: one FGD was conducted with a group of the team members from CIPARS and the other FGD was conducted with a group of end-users of CIPARS. The team members from CIPARS were selected using the following criteria: Employees of the PHAC with more than 75% of their task being part of the activities for CIPARS for more than 5 years. An email invitation to participate was sent to the team members of CIPARS who meet the inclusion criteria. Interested members were then invited to sign the consent form prior to participating in the FGD.

The end-users of CIPARS were selected using the following criteria: Active end-users of the knowledge and/or data produced by CIPARS for more than 5 years. A list of 653 end-users compiled by the team of CIPARS was used to identify the end-users who could provide a diverse set of perspectives across different areas of expertise (human and animal health) and organizations in Canada (public, private, non-profit, provincial, and federal). An invitation letter and the information and consent form were sent through email to a subgroup of 17 potential participants selected by the CIPARS.

Focus group discussions were conducted in December 2016; they lasted for 90 min, and were moderated in English by the principal researcher, Aenishaenslin. Key concepts that were explored included the definition of the integrated surveillance in the OH context, the expected outputs and outcomes from the integrated surveillance programs, and the objectives and needs of the evaluation. To develop a common definition of the integrated surveillance, the definition of OH surveillance proposed by Stärk et al. (19) was used as a starting point to be adapted: “the systematic collection, validation, analysis, interpretation of data, and dissemination of information collected on humans, animals, and the environment to inform decisions for more effective evidence- and system-based health intervention.” A full FGD guide is provided in Supplementary Material 1.

Focus group discussions were recorded and transcribed *verbatim*. The NVivo software (Version 12) was used to facilitate the classification and analysis of information about the definition and components of the integrated surveillance, expected outputs and outcomes, and the elements to consider for the evaluation. Convergent and divergent perspectives between and within the groups were examined. The lead author used this information to develop preliminary versions of definitions for key concepts, a generic logic model for OH integrated surveillance systems for AMU and AMR, the conceptual evaluation framework, and a semi-quantitative tool for measuring the level of integration.

### Phase 2: Workshop With CIPARS Team Members

In the second phase, a 3-h workshop was conducted in April 2017 with the same participants from the CIPARS team to discuss the preliminary findings and to agree on the next steps. Preliminary versions of the framework, including a logic model for a generic OH surveillance system for AMR and AMU, and the semi-quantitative measurement tool were first presented by the lead author. Participants were asked how well these models and tools represented their perspectives and evaluation needs. Feedback of the participants was collected in writing (Aenishaenslin) and used to revise the preliminary versions of these resources.

### Phase 3: Individual Questionnaires

In the third phase of the ISSE framework development, the team members of CIPARS were consulted for the last time in October 2017 using an individual, web-based questionnaire (Google forms software). The objective was to assess whether the final proposed items (definition of integrated surveillance, logic model, conceptual evaluation framework, and the semi-quantitative measurement tool) were perceived as relevant, complete, and clear by each participating team member, and whether they had any final comments and suggestions for improvements. All comments were examined by the research team and used for the final enhancement of each item. Final versions of the definition of the integrated surveillance, of the conceptual evaluation framework, and of the semi-quantitative measurement tool are presented in this paper. The logic model for the integrated surveillance system for AMU and AMR is described more in detail by Aenishaenslin et al. (13).

### Phase 4: Identification of Complementary Surveillance Evaluation Tools

**Figure 2:**
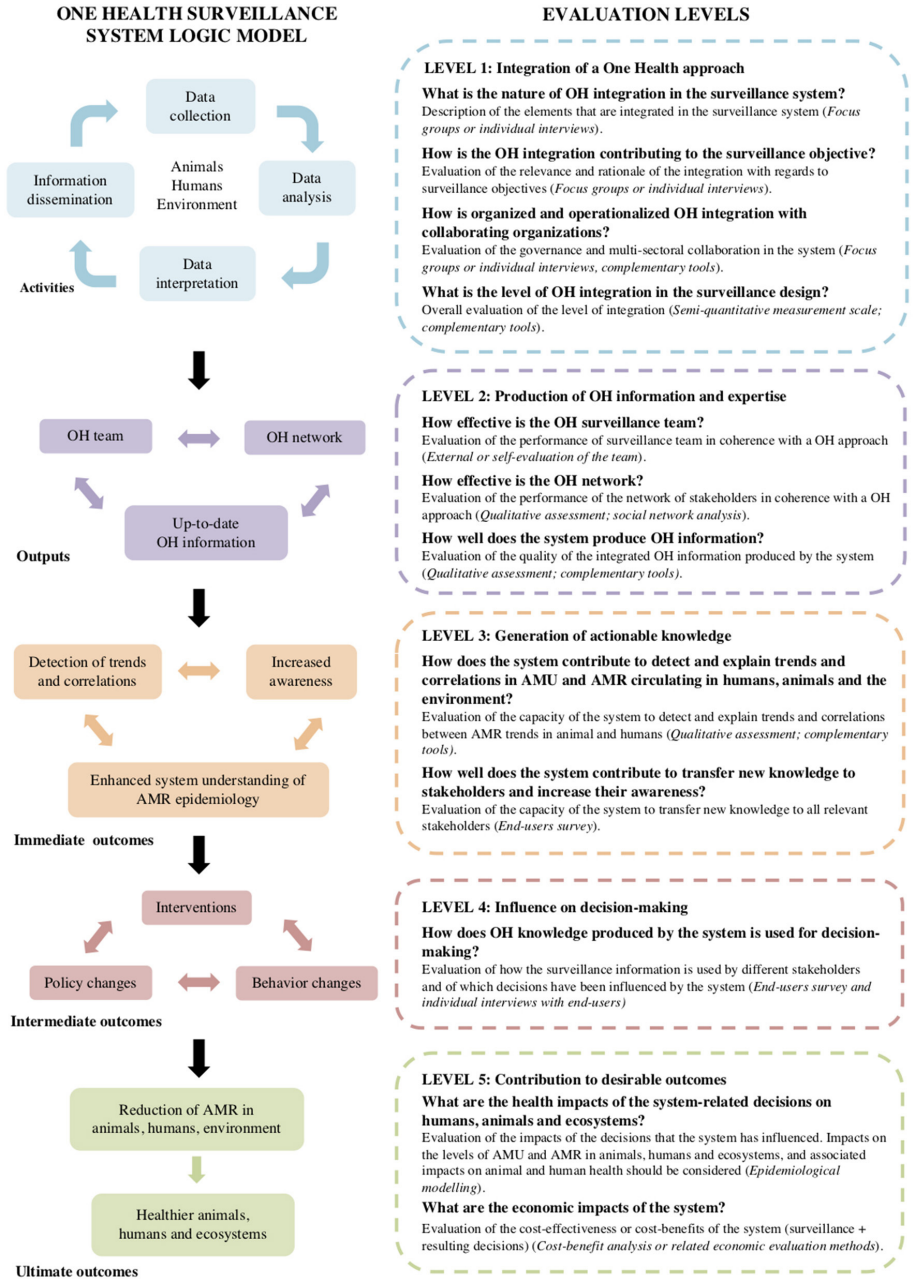
Final ISSE framework. The figure on the left describes a logic model for the integrated OH surveillance systems for AMR and the figure on the right describes five evaluation levels with the respective set of evaluation questions (suggested methodology for each question is presented in parenthesis).

The fourth and last phase of the study aimed to identify other evaluation tools that could be used at each evaluation level of the ISSE framework (Figure 2). When the current study was ongoing, several evaluation frameworks and tools were explored but none were designed to evaluate the added value of the OH integration into surveillance systems for AMU and AMR. Several evaluation tools were in development at that time and were potentially of interest to address some evaluation needs identified for this study. Consequently, a review of 12 evaluation tools focusing either on surveillance evaluation or on OH evaluation was conducted between January and December 2019, as a part of the research conducted with the CoEval-AMR Network (Convergence in evaluation frameworks for integrated surveillance of AM resistance and AM use), of which five of the authors are members (20). The following 12 tools were included: [1] Evaluation of collaboration for surveillance (EcoSurTool) (21), [2] Network for the Evaluation of OH Framework (NEOH) (22), [3] OH Assessment for Planning and Performance (OH-APP) (23), [4] The FAO Assessment Tool for Laboratory and AMR Surveillance Systems (ATLASS) (24), [5] Outil d'Analyse des Systèmes de Surveillance (OASIS) (25), [6] SuRveillance EVALuation framework (SERVAL) (26), [7] SurvTools (27), [8] Surveillance Evaluation Framework (SurF) (28), [9] The FAO Progressive Management Pathway for AMR (PMP-AMR) (29), [10] Joint External Evaluation tool (Second edition) (JEE) (30), [11] International Health Regulation core capacity monitoring framework (IHR) (31), and [12] The OIE Tool for the Evaluation of Performance of Veterinary Services (PVS) (32) (**Table 4**). For each tool, the general purpose, scope, process, and output were synthesized by the CoEval-AMR research team and all the evaluation items (questions or criteria) from these tools were extracted, examined, and attributed to one or more ISSE evaluation levels by three different analysts (Aenishaenslin, Mediouni, and Bennani) (20). Using this information, the relevant tools for each ISSE evaluation level were identified. Moreover, the ISSE framework was presented and discussed with 24 international experts, in surveillance and/or OH evaluation, who are members of the CoEval-AMR Network during a workshop conducted in October 2019, in order to collect feedback. This step allowed the identification of methodological gaps to be addressed in the future.

## Results

Eight of 10 invited team members from CIPARS and 7 of 17 invited end-users from CIPARS participated in the FGDs (Table 1). There was a lack of consensus between and within the team members and end-users from CIPARS, on how to define the integrated surveillance for AMU and AMR. Proposed explanations for this lack of consensus were that the essential components of an integrated surveillance system will differ depending on the objectives of the system, the targeted problem under surveillance (AMR in the food chain vs. AMR in general), and the expertise and background of the participants (animal vs. human health). Among the team members of CIPARS, integration was described as happening in different ways across the surveillance activities and was described as a continuum (multiple levels of integration) rather than a dichotomy (integrated vs. non-integrated systems). Three definitions of core integration concepts were developed for this study for “integrated surveillance,” “level of integration,” and “integrated surveillance system” (Table 2).

**Table 1:** Profile of individuals participating in focus group discussions (FGDs) (as self-reported).

**FGDs**	**Profiles**
CIPARS team (*n* = 8)	Management of CIPARS Analysis and integration of data Data analysis and stakeholder engagement in poultry Coordination of on-farm component operations Coordination of abattoir component operations Coordination of retail component operations Analysis of integrated AMU data Risk assessment
End-users (n = 7)	Expert in AMU and AMR in agriculture, provincial level Medical epidemiologist, provincial level Veterinary epidemiologist, provincial level Member of a committee for AMR stewardship in Canadian Agriculture and Veterinary Medicine Representative of an animal health organization, federal level Representative of a drug safety organization, federal level

**Table 2:** Definitions of core concepts relevant to integrated surveillance.

**Concept**	**Definition**
Integrated surveillance	Systematic collection, analysis, interpretation of data, and dissemination of information collected from different components of a system to provide a global, multidisciplinary, multiperspective understanding of a health problem and to inform system-based decisions across all relevant sectors [adapted from Stärk et al. (19)].
Level of integration	The level of integration of a surveillance system refers to the degree of intensity in the integration of different components within each surveillance activity (i.e., data collection, analysis, interpretation, and dissemination) across sectors, implemented to provide a global, multidisciplinary, and multiperspective understanding of a health problem and to inform system-based decisions.
Integrated surveillance system	An integrated surveillance system consists of a planned set of components that are organized and interconnected in order to achieve the objectives of the integrated surveillance, including its resources, infrastructures, activities, and internal and external factors.

### ISSE Framework

The final ISSE framework includes five evaluation levels, which correspond to the hierarchy of a generic logic model for the OH surveillance systems for AMU and AMR AMU (Figure 2). This hierarchy emerged from FGD and workshops conducted with the team members of CIPARS. The logic model illustrates the relationships between activities, direct outputs, and the immediate, intermediate, and ultimate expected outcomes of the surveillance system. Figure 2 presents the overall ISSE framework with questions at each evaluation level in relation to the logic model of the surveillance system. A description of each evaluation level and an overview of the proposed methods for each evaluation item are presented below.

### Level 1: Integration of a OH Approach

The first level aims at evaluating the integration of a OH approach across the different components of the surveillance system. A description of the nature of integration (What is integrated into the surveillance systems?), its relevance to the targeted surveillance objectives (How is OH integration contributing to achieving the surveillance objective?), and its organization and governance among collaborators and collaborating organizations (How is the integration between the collaborators organized and operationalized?) are the key aspects to be addressed at this level.

The above three questions can be addressed using a descriptive qualitative methodology. The evaluator can use focus groups or individual interviews to address these questions with key informants from the surveillance team, external end-users, and AMR/AMU experts. The information provided in response to this first set of questions can be then used to assess the overall level of integration into the system, using the semi-quantitative scale presented in Table 3. This scale defines six levels of OH integration into the surveillance design for data collection, analyses, interpretation, and dissemination, with respect to the type of information that is integrated, the integration into operations and processes (e.g., standardization of measures), and the integration of multiple institutions, disciplines, and perspectives in coherence with a OH approach.

**Table 3:** Measurement scale for One Health (OH) integration levels in the surveillance design for AMR in foodborne bacteria.

	**0**	**1**	**2**	**3**	**4**	**5**
Data collection	The surveillance system (SS) does not collect data from sources other than humans.	The SS collects AMR data (passive or active) in one bacteria species, from one animal source (other than human) that is one animal species/commodity at one collection point.	The SS collects AMR data (passive or active) in >1 bacterium species OR from >1 animal source (more than one animal species or more than one collection point).	The SS collects AMR data (passive or active) in >1 bacterium species, AND from >1 animal source (more than one animal species or more than one collection point).	The SS collects AMR data (passive or active) in >1 bacteria species from >1 animal source including retail food AND it collects data from at least one collection point in the environment (outside farm environment) OR on AMU in ≥1 animal species and humans.	The SS collects AMR data (passive or active) in >1 bacteria species from >1 animal source including retail food AND from at least one collection point in the environment (outside farm environment) AND it collects data on AMU in ≥1 animal species and humans.
Data analysis	Animal and human data analyses are done separately for each data source, by different analysts in ≥2 organizations, and analysis are not standardized to allow comparisons between the components.	Animal and human data analyses are done separately for each data source, by different analysts in ≥2 organizations, but reporting of analyses is standardized to allow the end-users to compare between the components of their interest. There is no formal structure/team/committee in charge of comparing the spatial and temporal trends between animal and human components.	Animal and human data analyses are done separately for each data source, by different analysts in ≥2 organizations, but reporting of analysis is standardized AND there is a formal inter-organizational structure/team/committee in charge of comparing and reporting the spatial and temporal trends between the animal and human components. Integrated analyses are mostly restricted to descriptive analysis and they do not include formal statistical comparisons of AMR/AMU levels in animals and humans.	Animal and human data analyses are done separately for each data source, by different analysts in the same organization. Analyses are standardized AND there is ≥1 person in charge of comparing and reporting spatial and temporal trends in AMR between the animal and human components. Integrated analyses are mostly restricted to descriptive analysis and don't include formal statistical comparisons of the AMR/AMU levels in animals and humans.	Animal and human data analyses are done separately for each data source, by different analysts in the same organization. Analyses are standardized AND there is ≥1 person in charge of comparing and reporting the spatial and temporal trends in AMR between the animal and human components. Integrated analyses include formal statistical comparisons of the AMR/AMU levels in animals and humans.	Animal and human data analyses are done conjointly by a team of analysts. Spatial and temporal trends are compared systematically with formal statistical testing. Integrated analyses also include multivariable statistical approaches or modeling to quantify the relationships between the AMR/AMU levels in animals and humans.
Data inter-pretation	There are no formal integrated analysis (level of integration = 0 or 1 for data analysis).	Integrated analyses are interpreted by one person with specific expertise in AMR/AMU epidemiology in general or in one animal species.	Integrated analyses are interpreted by a team of several people with the same specific expertise to AMR/AMU epidemiology in general or in one animal species.	Integrated analyses are interpreted by a team of several people with multispecies expertise regarding AMR/AMU epidemiology (include experts from ≥2 animal species).	Integrated analyses are interpreted by a team of several people with multispecies/multidisciplinary expertise regarding AMR/AMU epidemiology [include experts from ≥2 animal species AND ≥2 people with expertise in another relevant field for AMR surveillance (e.g., pharmacist, economist, and social science experts)].	Integrated analyses are interpreted by a team of several people with multispecies/multidisciplinary/multiperspective expertise regarding AMR/AMU epidemiology (include experts from ≥2 animal species AND ≥2 people with expertise in another relevant field AND ≥2 external collaborators/stakeholders from different sectors).
Information dissemination	Information for animals is reported to animal health stakeholders and information for humans is reported to human health stakeholders.	Integrated information is reported but it is done separately to animal and human stakeholders by several organizations, without inter-organizational coordination/harmonization.	Integrated information is reported separately, mainly to animal and human stakeholders, by >1 organization, but efforts are done to harmonize the reporting in a comparable format (multiple means of dissemination are used, for example, through several annual reports).	Integrated information is reported conjointly to animal and human stakeholders by >1 organization (multiple means of dissemination are used).	Integrated information is reported conjointly to animal and human stakeholders by one organization, but only one general mean of reporting is used for all end-users (e.g., annual report).	Integrated information is reported conjointly to animal and human stakeholders by one organization AND the different means of dissemination are adapted to different end-users.

### Level 2: Production of OH Information and Expertise

At level 2, the capacity of the surveillance system to produce OH information and expertise is assessed. Three main outputs were defined in the logic model and they can be evaluated at the following levels: [1] the performance of the OH surveillance team (the operational team), [2] the performance of the OH network of stakeholders who are united by the surveillance system, and [3] the capacity of the system to produce up-to-date OH information on AMU and AMR. The OH team is defined here as the interdisciplinary team that creates to integrate the information produced by the surveillance system. Depending on how the surveillance system is governed, the team may be composed of individuals within one or several organizations that are involved in the surveillance system. For example, CIPARS is operated by one organization, the PHAC, and consequently has one formal team responsible for the collection and integration of information. In contrast, other AMU and AMR surveillance systems are operated collaboratively by two or more collaborative organizations, with each organization responsible for different components of the system (e.g., monitoring of animals and of humans). In these cases, the OH team can be composed, for example, of members of the organizations that are mandated to participate in an intersectoral committee in charge of the integration of the information produced by the different surveillance components. To assess the performance of the OH team, an external or self-evaluation process can be undertaken using group discussions and through individual interviews with the team members.

Similarly, the OH network of stakeholders, which shares information, thanks to the existence of the surveillance system, can be evaluated at this level. A qualitative assessment of the representation in this network of relevant expertise and organizations covering the different fields of OH, and an evaluation of the role and contribution of each can be undertaken. Although more time-consuming, advanced evaluation and monitoring of an evolving network could also be evaluated using the social network analysis (33).

Finally, the quality of the OH information produced by the system can be evaluated by examining the types of data collected by the system in relation to the OH surveillance objectives. For example, the evaluator can assess if all necessary data are collected from different animal species, humans, and in the environment in order to produce information with a OH perspective.

### Level 3: Generation of Actionable Knowledge

Level 3 aims to evaluate how the combination of OH information and its interpretation by the OH team and its dissemination to the OH network contributes to the generation of actionable knowledge at the human-animal-environment interface. In contrast to the evaluation of OH information produced by the system, which focuses on the quality of the data in itself and the relevance of the information captured by the data, this level aims to evaluate what is being done with this information in order to make it actionable. Two elements that are particularly important at this stage are as follows: [1] an evaluation of the capacity to detect and explain trends in the AMR circulating between humans, animals, and the environment, which then lead to a better understanding of the epidemiology of this complex problem, and [2] an evaluation of the capacity of the system to translate and transfer this information to relevant stakeholders in order to increase their awareness of AMR issues at the human-animal-environment interface. Of central interest here is to examine how well the dissemination activities reach all the necessary stakeholders, particularly those who are able to act, develop, and implement effective and sustainable OH interventions.

In terms of methods, the first elements (detection and explanation of trends and correlation) can be assessed by examining the surveillance design and more specifically, the types of analyses that are being conducted by the surveillance team. The last element (knowledge translation and transfer to increase awareness) can be assessed using an end-user survey.

### Level 4: Influence on Decision-Making

Level 4 aims at evaluating how the knowledge generated by the integrated system is concretely used for decision-making and how it contributes to actions and changes when needed. This includes evaluating how the knowledge generated contributes to decisions regarding [1] interventions to be implemented (for example, sanctions if higher levels of AMU are detected in a particular animal species or human setting), [2] policy changes (for example, restricting the use of some types of AM in agriculture by public or private institutions), and [3] individual behaviors that are not necessarily linked to formal policy changes. For example, prescribing practices by veterinarians or physicians may change only if the information reaches them and only if they are able to understand and use this new knowledge to change their own behaviors. Even though we recognize that these decisions will be as well influenced by other factors external to the surveillance system (for example, demand from consumers and media pressure, among others), we believe that understanding the capacity of the system to influence these changes is an important indicator of the performance and impacts of the system.

End-user surveys, in combination with individual key informant interviews, may be used as a method at this level. The survey can provide quantification of the main ways the knowledge is used and of the perceived influence of the surveillance system on higher level decisions, such as policy changes. Individual interviews can provide rich, nuanced, and additional information to better understand the strengths and weaknesses of the system in its capacity to influence decision-making.

### Level 5: Contribution to Desirable Outcomes

Finally, the impacts of the decisions that can be attributed to the OH integration into the surveillance system can be evaluated at Level 5, including impacts on the reduction of AMU and AMR and their consequences to the health of humans, animals, and the ecosystem. Methods for evaluating the impacts of OH integration into surveillance systems will differ depending on the types of impacts that need to be evaluated. Epidemiological modeling is a methodological approach to consider at this step (34).

The economic efficiency of the whole OH mitigation system (i.e., the integration of OH in the surveillance system and in the implemented interventions) can also be evaluated at this level. One way to assess these impacts would be to conduct a cost-benefit analysis (35). The inputs and effects associated with an integrated surveillance approach can be contrasted with the inputs and effects associated with a hypothetical unisectoral approach. Costs associated with the integration can be estimated as the total operating costs that are needed for operating the surveillance system (surveillance costs), including the cost of integrating human, animal, and environmental data, and the costs related to implemented interventions (intervention costs). Surveillance costs include the costs of collecting and analyzing human, animal, and environmental data (sampling, laboratory testing, storage, equipment, permanent and temporary staff, and travel) and the costs associated with the dissemination and reporting of the surveillance information (communications, meetings, documents, publications, and staff). Intervention costs include the costs associated with the implementation of any intervention, policy, or behavior change attributed to the outcomes of the integrated surveillance (for example, using alternative AM in animal productions and potential production losses, and costs of communication campaigns). The costs associated with a unisectoral scenario could be estimated separately as the operating costs of surveillance in sectors, without considering costs or savings of the integrating animal and/or environmental data and the costs of implementing the additional actions that would not have been implemented due to lack of integration. The benefits of the integrated surveillance approach can be estimated as the averted costs that would have arisen under the unisectoral scenario and the difference in the outcomes produced (e.g., reduction in the duration of disease, complications, or severity in humans or animals related to the failure of the AMR treatment; and reduction in the AMR-related mortality in animals and humans).

### Relevance of Surveillance Evaluation Tools

Table 4 presents an overview of the purpose of each tool and the evaluation levels of ISSE for which they could provide information. None of the identified tools was specifically developed for the evaluation of OH integration into AMR and AMU surveillance systems, and none covers all the information required at all the five levels of the ISSE framework. Among the 12 tools examined, only the PMP–AMR and the ATLASS tools were specifically developed for the AMR and AMU surveillance systems, and the NEOH tool was specifically developed for the OH evaluation. Of interest, the EcoSurTool is dedicated to the evaluation of collaboration with surveillance systems and could be used to evaluate the aspects of OH integration (Level 1) in the integrated surveillance systems for AMR and AMU. Finally, even though the importance of measuring impacts is mentioned in several tools, none includes specific guidance for evaluating the global health and economic impact of OH integration into AMR and AMU surveillance systems.

**Table 4:** General purpose of available evaluation tools (20) and their relevance for each ISSE evaluation level.

**Tool (Reference)**	**Year of publication**	**General purpose**	**Levels of evaluation**
ECoSur (21)	2019	Evaluation tool for the organization, functioning, and functionalities of collaboration taking place in a multisectoral surveillance system.	1
NEOH (22)	2018	Tool to assess the extent to which the six aspects of knowledge integration are implemented in an initiative or a surveillance system, including thinking, planning, transdisciplinary working, sharing, learning, and systemic organization.	1, and parts of 2 and 4
OH-APP (23)	2018	Tool to assess the maturity of multisectoral coordination mechanism and to provide data for decision-making that would enhance the organizational capacity and OH performance.	Parts of 1
ATLASS (24)	2016	Tool developed by FAO to help identify targets to improve the national AMR surveillance systems in the food and agriculture sectors.	Parts of 1, 2, 3 and 4
OASIS (25)	2011	Tool for the evaluation of surveillance systems in animal health, food safety, and plant health.	Parts of 2, 3, and 5
SERVAL (26)	2015	Evaluation framework for the comprehensive evaluation of single surveillance components (activities) or the entire surveillance programs.	Parts of 2, 3, and 5
SurvTool (27)	2018	Surveillance evaluation tools and framework for providing step-by-step guidance for the evaluation of surveillance (all sectors).	Parts of 2, 3, and 5
SurF (28)	2016	Surveillance evaluation tools and framework for the animal, plant, environment, and marine sectors.	Parts of 2, 3, and 5
PMP-AMR (29)	2019	Tool developed by the FAO to provide guidance to countries for developing and operationalizing their multi-sector OH National Action Plans on AMR through a stepwise approach.	Parts of 2, 3, and 4
JEE (30)	2018	Tool developed by the WHO to establish country-specific status and progress in achieving the targets defined by the International Health Regulations (IHR).	Parts of 2, 3, and 5
IHR (31)	2015	Tool developed for the assessment of capacities at the human-animal interface in the IHR Monitoring Framework.	Parts of 1, 2, and 3
PVS (32)	2013	Tool developed by Organization for Animal Health (OIE) for the evaluation of the application of its standards and guidelines.	Parts of 2 and 3

## Discussion

This article describes a conceptual framework for the evaluation of OH integration into surveillance systems for AMU and AMR. The proposed framework is not meant to replace the existing evaluation tools for surveillance systems. It should be considered as an overarching conceptual base for structuring the development of an evaluation protocol, which must be adapted according to the needs of an evaluator.

The five evaluation levels were determined with respect to the hierarchy in the expected chain of events that emerged from the discussions and link the surveillance activities and outputs to the immediate, intermediate, and ultimate outcomes of the system.

The complementarity of the existing evaluation tools with the five evaluation levels was assessed. Even if not specific to AMR and AMU surveillance, some new OH evaluation tools have been developed in the last 5 years and they can be used to assess a part of the OH integration, such as the ECoSurTool, developed by Bordier et al. (21). This tool is designed to evaluate the collaboration between factors involved in different surveillance components and comprises a group of several attributes and criteria that can be evaluated and translated into quantitative scores. Another recent work conducted by the Network for the Evaluation of One Health (NEOH) has developed and tested a methodological approach to measure the degree of OH implementation in projects or programs (22). Standardized questionnaires can be used to score the six dimensions of OH, such as thinking, planning, working, learning, sharing, and systemic organization. These metrics can then be integrated to quantify the strength of the OH (the “OH-ness”) of initiatives. Although these metrics were not developed specifically to evaluate the OH integration into surveillance systems, they can be applied to surveillance systems as a complementary approach. Case studies of the application of these evaluation tools have been published recently (36, 37).

Overall, linking the existing tools to the specific evaluation levels and questions of ISSE framework was difficult, most likely because none of these tools was specifically designed to evaluate the OH surveillance systems. Some elements of importance for Levels 2 and 3 are included in the evaluation tools for several surveillance systems, but proposed metrics and methods were only partially relevant for most evaluation questions included in the ISSE framework.

Most surveillance evaluation frameworks examined in this study have been developed to assist the evaluators in choosing groups of surveillance attributes to evaluate, such as sensitivity and timeliness (18, 38). Some of these surveillance attributes can be used to evaluate the different components of AMR and AMU surveillance systems but they need to be adapted to be useful for evaluating the OH integration into the system. For example, instead of estimating the sensitivity of the surveillance system as prescribed in most existing surveillance evaluation frameworks, the gain in the sensitivity of a surveillance system with the addition of other data sources from animal origins could be used to assess the capacity of the system to detect trends in AMR circulating at the human-animal interface. We also observed an overlap and complementarity in the available tools, which may cause confusion among occasional users. In order to provide guidance to evaluators who need to choose suitable evaluation tools to evaluate surveillance systems for AMR and AMU, the CoEval-AMR Network has developed guidance and a decision-tool that are available for users (20).

The only tool dedicated specifically to AMR surveillance systems that was examined in this study is the ATLASS tool, developed recently by the Food and Agriculture Organization of the United Nations (24). The ATLASS is a user-friendly evaluation tool that assists countries in the development and improvement of their national AMR surveillance systems in the food and agriculture sectors and addresses some elements of importance for the integration of a OH approach. However, it mostly addresses the animal components of surveillance systems and does not provide guidance on how to evaluate the integration with other sectors (human and environment).

This study also identified other elements that are rarely included in the evaluation of surveillance systems. One example is the interdisciplinary team and its network, which were considered as major (and yet under-evaluated) drivers of surveillance effectiveness by the team of CIPARS. Teamwork has been recognized as an important component of effective organizations in different settings, including in health systems (39), and has been recognized as an important component of successful OH initiatives (40, 41). However, the evaluation of this element has been mostly absent in the evaluation tools for surveillance systems, currently available. We believe that an in-depth examination of evaluation tools used in other disciplines not targeting surveillance or One Health evaluation (such as management, social and economic sciences) may offer opportunities to improve surveillance evaluation in general and should be done in the near future.

Finally, this study also underlined an important remaining methodological challenge: the evaluation of surveillance impacts for AMU and AMR. Previous research has led to the development of concepts and methods to evaluate the economic impact of OH surveillance (35, 42). However, we could not find any other studies that have quantified the health and economic impacts of integrated surveillance systems for AMR and AMU or adapted methods for such quantification.

In the framework for the economic evaluation of OH surveillance designed by Babo Martin et al. (35), the costs and benefits of the targeted OH surveillance system are compared to the costs and benefits of a past or hypothetical unisectoral (i.e., not integrated) surveillance system. The costs and benefits of interventions that were implemented because of OH surveillance information are also included, in line with the conceptualization of surveillance benefits by Häsler et al. (43). Using this framework, two case studies were conducted in the European context and they were found with conflicting results. One of the studies measured the cost-effectiveness of the surveillance for *Campylobacter* in Switzerland and it did not find cost and health benefits of OH integration over their study period (44). A second study measured the costs and benefits of the OH surveillance for the West Nile virus in Northern Italy and revealed important cost savings (more than one million Euros) due to avoided tests on blood units when using animal surveillance data for decision-making (45). These studies demonstrated the feasibility of quantifying the value of OH surveillance and led to calls for more economic evaluation in other contexts. Complex OH surveillance systems, such as surveillance systems for AMR and AMU may not produce direct economic benefits in the short-term because they may not lead immediately to specific interventions. Intellectual and social capital benefits, which are difficult to capture through quantitative metrics in the short-term, may lead to large benefits in the long-term (46). However, these studies could be useful as a starting point for structuring the evaluation of health and economic impact of the integrated OH surveillance systems for AMR and AMU.

The definition of integrated surveillance that was adopted for this study was adapted from Stärk et al. (19), but differs slightly from a more recent definition of OH surveillance that has been proposed by Bordier et al. (47). The authors of this study define a OH surveillance system as “a system in which collaborative efforts exist between at least two sectors (i.e., human health, animal health, plant health, food safety, wildlife, and/or environmental health) at any stage of the surveillance process, to produce and disseminate information with the purpose of improving an aspect of human, animal, or environmental health” (47). Our definition does not explicitly include collaboration as an essential component of OH surveillance, and this difference can be explained by the particular governance model of CIPARS. The operation of CIPARS, including the surveillance activities conducted in its animal components, is centralized in one organization (PHAC), although the program works in close collaboration with numerous partners, including the Canadian Food Inspection Agency, Health Canada, and the animal production industry. This is in contrast with other systems in which the animal and human components are coordinated and funded by different organizations, usually by the agriculture and health sectors, respectively. Consequently, even though CIPARS adopts a OH approach and is highly integrated, its operationalization does not require the same level of inter-organizational collaboration.

This study has limitations. First, only a small group of end-users were invited to participate in the FGD, and their perspective may not reflect the perspective of the larger group of end-users of CIPARS. Second, the ISSE framework was developed in partnership with CIPARS and may better reflect the evaluation needs of a mature, highly integrated surveillance system for AMU and AMR, than the evaluation needs of more recent, or less integrated surveillance systems. In particular, external experts from the CoEval-AMR network correctly underlined that the availability of longitudinal data for evaluating surveillance impacts as suggested at Level 5, as well as the time and resources required for conducting the full evaluation process (at all levels) will constitute important challenges associated with this approach (36). The ISSE framework was developed to be comprehensive, but evaluators can choose to focus on one or two evaluation levels depending on their needs, rather than on all five evaluation levels. For example, we suggest focusing on evaluation levels 1 and 2 in the first 3–5 years of implementation for new or recently integrated surveillance systems. More will be learned about the added value of the OH surveillance for AMU and AMR globally once multiple case studies are completed, as it will allow to explore the relationship between the higher level of OH integration (at Levels 1 and 2) and surveillance outcomes (at Levels 3 to 5).

## Conclusions

Evidence of the added value of OH surveillance for AMR and AMU is needed to inform the development of effective and efficient systems worldwide. The proposed conceptual framework can guide the development of an evaluation protocol to start compiling this evidence. This study also calls for more research on methods and tools to evaluate the health and economic impacts of the OH surveillance systems in general. The next steps include applying the framework to evaluate various surveillance systems, including CIPARS, in order to appreciate its applicability and usefulness under real conditions and different contexts.

## Data Availability Statement

The original contributions presented in the study are included in the article/Supplementary Material, further inquiries can be directed to the corresponding author/s.

## Ethics Statement

The study protocol involving human participants was reviewed and approved by McGill Institutional Review Board (IRB Study Number A09-E61-16B). The participants provided their written informed consent to participate in this study.

## Author Contributions

CA collected data, coordinated the study phases, and wrote the first version of the manuscript. CA, BH, SM, HB, and EJP contributed in the data analysis. All authors contributed to the research design, data interpretation, framework development, writing of the manuscript, and approved the final version of the manuscript.

## Conflict of Interest

The authors declare that the research was conducted in the absence of any commercial or financial relationships that could be construed as a potential conflict of interest.
