# Supplementary material for: Evaluating the Integration of One Health in Surveillance Systems for Antimicrobial Use and Resistance: A Conceptual Framework
Source: Front Vet Sci. 2021 Mar 24;8:611931. doi: 10.3389/fvets.2021.611931 (PMC8024545; doi:10.3389/fvets.2021.611931)
Supplement: Supplementary file 1 [file Data_Sheet_1.pdf]

Integrated One Health surveillance system evaluation, Aenishaenslin C. et al. 2020

Supplementary material 1: Focus Group Discussion interview grid

1. Can you tell us your first name and explain the role that you play in your organization in relation to integrated surveillance?
2. There is a global consensus among experts that integrated surveillance systems are necessary to face the problems of zoonosis and AMR. However, there is no consensus on the definition and essential components of an integrated surveillance system at the human-animal interface. Is the definition of ISS clear for you and for your organization?
3. What is an integrated surveillance system for problems at the animal-human interface?
4. What should be the objectives of an integrated surveillance system in opposition/in addition to a non-integrated surveillance system for AMR?
5. What is/should be the added value of ISS for AMR?
6. Which components are essential to include in order to have an integrated surveillance system for AMR, regarding the collection and analysis of data, and the interpretation and dissemination of information generated?
7. What is an effective ISS for AMR?
8. What is an economically efficient ISS for AMR?
9. Is 'integration' always necessary for surveillance of zoonoses and AMR? In which situations would integration not have an added value to a surveillance system? Can you give examples of unnecessary or exaggerated levels of integration?
10. What are the challenges of evaluating an integrated surveillance system for AMR?
11. What are the challenges of using information from an integrated surveillance system for AMR?
